# Nuclear PCGF3 inhibits the antiviral immune response by suppressing the interferon-stimulated gene

**DOI:** 10.1038/s41420-024-02194-x

**Published:** 2024-10-05

**Authors:** Gula Da, Junmin Wang, Jing Shang, Cuiping Xun, Yang Yu, Yong Wang, Ning Tie, Hongbin Li

**Affiliations:** 1 Department of Rheumatology and Immunology, The Affiliated Hospital of Inner Mongolia Medical University, Hohhot, China; 2 Inner Mongolia Key Laboratory for Pathogenesis and Diagnosis of Rheumatic and Autoimmune Diseases, Hohhot, China; 3 Department of Immunology, Institute of Basic Medical Sciences, Chinese Academy of Medical Sciences and Peking Union Medical College, Beijing, China

**Keywords:** Innate immunity, Cell biology

## Abstract

Type I interferon (IFN-I) plays a crucial role in the antiviral immune response and inflammatory autoimmune diseases by inducing the expression of IFN-stimulated genes (ISGs). Hence, the regulation of ISG expression is fundamental for maintaining immune homeostasis. In this study, we found that PCGF3 negatively regulates the antiviral response by suppressing the expression of ISGs. The deficiency of PCGF3 in innate immune cells results in an augmented expression of ISGs in response to IFN-I stimulation. Mechanistically, PCGF3 is recruited to interferon-stimulated response elements (ISREs) region in an IFN-dependent way, precluding STAT1 from binding to the ISG promoter and diminishing ISRE activity. Additionally, we observed a negative correlation between decreased PCGF3 expression and elevated ISG expression in peripheral blood mononuclear cells (PBMCs) of patients with dermatomyositis (DM). Our findings clarified the epigenetic regulatory role of PCGF3 in inhibiting the excessive expression of ISGs induced by IFN-I under pathological circumstances.

## Introduction

Type I interferons (IFN-I) (IFN-α and IFN-β) are produced by most cell types through intracellular and endosomal nucleic acids in response to stimulation by pattern recognition receptors [1–3]. IFN-I is involved in several biological processes that play a role in numerous autoimmune diseases, including systemic sclerosis (SSc) [4, 5] systemic lupus erythematosus (SLE) [5–7] and dermatomyositis (DM) [7–11]. The IFN signature in the peripheral blood of patients with autoimmune diseases has been studied to predict or assess disease activity [12, 13]. Recent studies have investigated the efficacy of JAK inhibitors in the treatment of a number of autoimmune and autoinflammatory diseases [14–16], suggesting a causal relationship between elevated levels of IFN-I and the pathogenesis of these diseases. Studying the regulatory mechanisms underlying the expression of IFN-stimulated genes (ISG) will help us to understand inflammatory diseases induced by IFN-I.

IFN-I treatment initiates tyrosine phosphorylation and enzymatic activation of both JAK1 and Tyk2 [1, 17]. Subsequently, the phosphorylation of STAT1 and STAT2 leads to heterodimerization, interaction with IRF9, and the formation of ISGF3 [1, 17]. Once translocated to the nucleus, this complex binds to conserved DNA elements within the promoter of IFN-responsive genes and induces the transcription of the ISRE of more than 300 interferon-stimulated genes (ISGs), such as ISG15, OAS1-3, IFIT1-3, MX1-2, and STAT1-2 [18, 19], which play a crucial role in antiviral activity [1, 20, 21]. A delicate balance between enhancing and suppressing mechanisms for ISGs allows for an effective antiviral response while avoiding deleterious effects on host tissues [22, 23]. There is increasing evidence that E3 ubiquitin ligases (E3s) regulate innate and adaptive immunity by ubiquitylating the proteins involved in the immune response [24, 25]. RING E3 is a major type of E3 and is characterized by its RING domain [26, 27]. During ubiquitination, the RING domain of RING E3 ligases binds to the E2 conjugation enzyme, and Ub is directly transferred from E2 to the substrate [28]. E3s up- or down-regulates the activity of specific members of the interferon signalling pathway by ubiquitylating modifications [29]. Different ISGs may enhance or inhibit the expression of E3s, thus forming a feedback regulatory mechanism [30, 31]. However, it remains to be determined which members of the nuclear E3s family are able to directly regulate the transcription of the ISGs.

Members of the polycomb group ring finger family (PCGF1-6) are critical components of polycomb repressive complex 1 (PRC1), which plays an important role in transcriptional repression by catalyzing the monoubiquitination of histone H2A [32]. Specifically, PCGF proteins bind to RING1A/B via the RING domain to enhance its enzymatic activity [33]. The RING domain of PCGFs has no E3 ubiquitin ligase activity, but it can bind directly to E2, thereby stabilizing the ubiquitination activity of RING1A/B [33]. In contrast to the canonical role of PRC1 in repressing genes, PCGF3 mainly acts as a transcriptional repressor or activator, regulating the expression of many genes involved in mesoderm differentiation [34]. However, whether PCGF3 mediates ISG expression remains unclear.

Here, we found that after IFN-I treatment, PCGF3 was recruited to the ISRE, preventing p-STAT1 binding to chromatin and repressing the transcription of ISGs. Our study provides insight into the negative regulation of ISG expression by IFN-I and suggests the potential importance of PCGF3 in the diagnosis or treatment of clinical diseases, ranging from inflammatory to autoimmune diseases.

## Results

### PCGF3 inhibits antiviral response

**Fig. 1:**
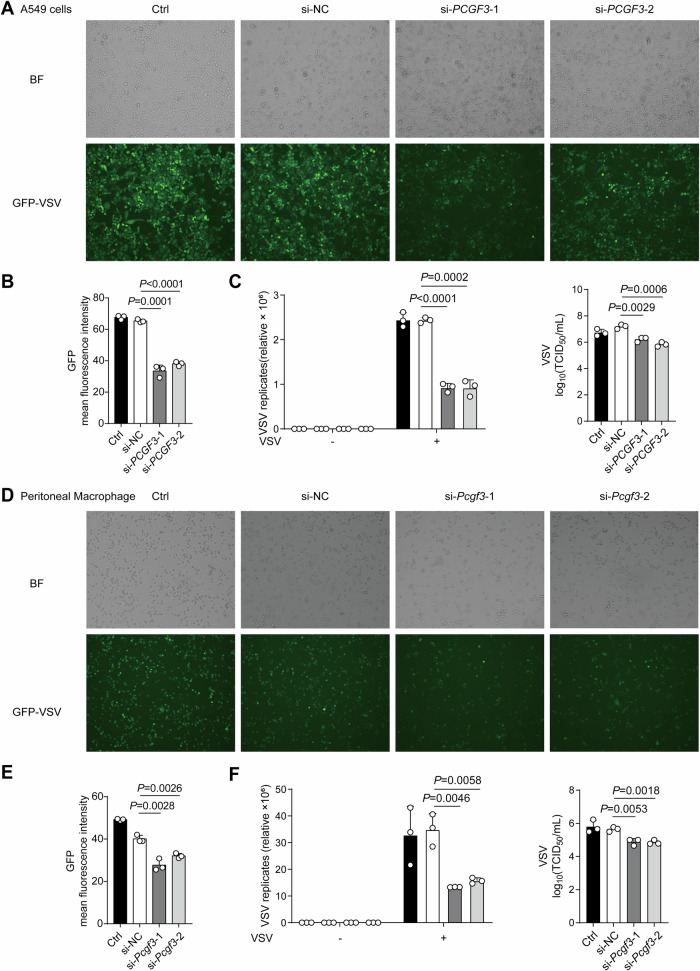
PCGF3 inhibits antiviral response in cells. **A** Typical images of 3 biological replicates of A549 cells without treatment (Ctrl) and A549 cells transfected with si-*PCGF3* (*si-PCGF3*-1 and *si-PCGF3*-2) or si-NC for 48 h, and then infected with GFP–VSV (MOI = 1) for 8 h. BF: Bright Field. **B** The GFP mean fluorescence intensity of 3 biological replicates in (**A**). **C** qRT-PCR analysis of VSV replicates (left) and cell culture supernatant viral titers (right) in A549 cells without treatment (Ctrl) and A549 cells transfected with si-*PCGF3* (*si-PCGF3*-1 and *si-PCGF3*-2) or si-NC for 48 h, and then infected with VSV (MOI = 1) for 8 h. **D** Typical images of 3 biological replicates of mouse peritoneal macrophages without treatment (Ctrl) and mouse peritoneal macrophages transfected with si-*Pcgf3* (*si-Pcgf3*-1 and *si-Pcgf3*-2) or si-NC for 48 h, and then infected with GFP–VSV for 8 h. BF: Bright Field. **E** The GFP mean fluorescence intensity of 3 biological replicates in (**D**). **F** qRT-PCR analysis of VSV replicates (left) and cell culture supernatant viral titers (right) in mouse peritoneal macrophages without treatment (Ctrl) and mouse peritoneal macrophages transfected with si-*Pcgf3* (*si-Pcgf3*-1 and *si-Pcgf3*-2) or si-NC for 48 h, and then infected with VSV (MOI = 1) for 8 h. Images in (**A**) and (**D**) were captured by EVOS system (Thermofisher). Mean fluorescence intensity was measured by ImageJ. Data are shown as mean ± SD of *n* = 3 biological replicates (1 field for each repeat experiments, **A** and **D**), two-tailed unpaired Student’s t-test (**B**, **C**, **E** and **F**).

To determine whether PCGF3 regulates the antiviral immune response, we transfected the human lung adenocarcinoma A549 cells with small interfering RNAs (siRNAs) targeting the *PCGF3* gene (si-*PCGF3*-1 and si-*PCGF3*-2). Knocking down PCGF3 with siRNA significantly reduced PCGF3 expression (Fig. S1A) and attenuated the fluorescence of GFP (GFP-VSV) (Fig. 1A, B). Consistently, knockdown of PCGF3 increased the expression levels of ISGs (*ISG15, IFIT1, OAS2, MX1* and *IRF7)* and *IFNB1* (Fig. S1B), and decreased the VSV replicates (RNA level and virus titer) after VSV infection (Fig. 1C). We observed similar effects in mouse peritoneal macrophages (Fig. 1D–F and Fig. S1C, D). The overexpression of PCGF3 also increased the fluorescence of GFP-VSV in RAW264.7 cells (Fig. S1E). These results suggest that PCGF3 suppresses the antiviral immune response in innate immune cells from human and mouse.

### PCGF3 inhibits ISG expression

**Fig. 2:**
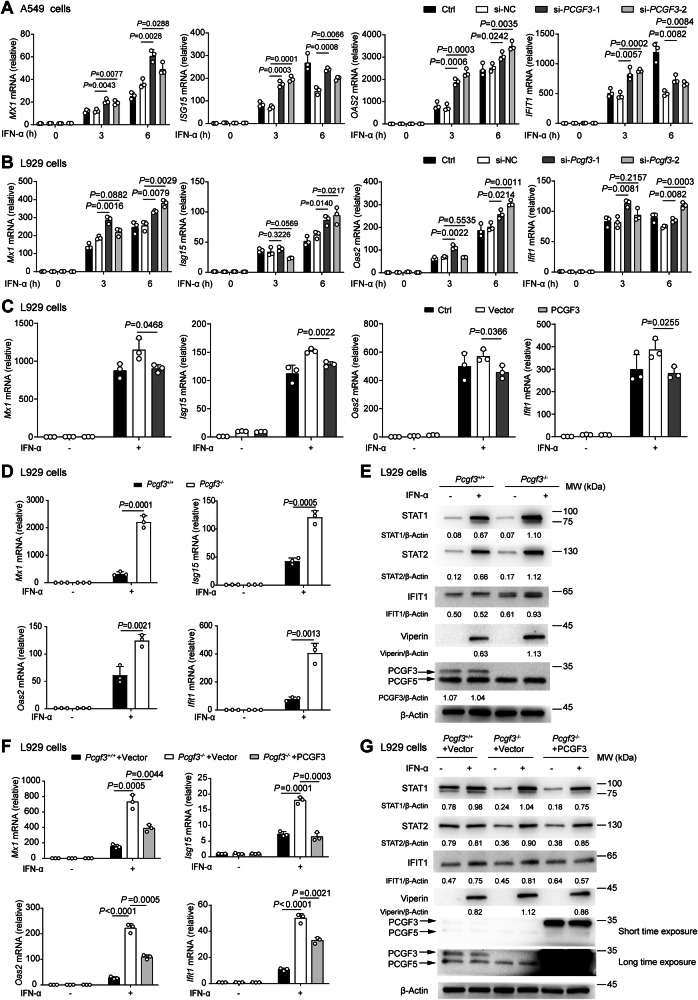
PCGF3 inhibits the expression of ISGs. **A** qRT-PCR analysis mRNA of expression of *MX1*, *ISG15*, *OAS2* and *IFIT1* in A549 cells without treatment (Ctrl) and A549 cells transfected with si-*PCGF3* (*si-PCGF3*-1 and *si-PCGF3*-2) or si-NC for 48 h, and then treated with human IFN-α (10 ng/mL) for the indicated times. **B** qRT-PCR analysis mRNA of expression of *Mx1*, *Isg15*, *Oas2* and *Ifit1* in L929 cells without treatment (Ctrl) and L929 cells transfected with si-*Pcgf3* (*si-Pcgf3*-1 and *si-Pcgf3*-2) or si-NC for 48 h and then treated with mouse IFN-α (50 ng/mL) for the indicated times. **C** qRT-PCR analysis mRNA of expression of *Mx1*, *Isg15*, *Oas2* and *Ifit1* in L929 cells without treatment (Ctrl) and L929 cells transfected with the empty vector (Vector) or the PCGF3 overexpression vector (PCGF3) for 24 h, and then treated with or without mouse IFN-α (50 ng/mL) for 6 h. **D** qRT-PCR analysis mRNA of expression of *Mx1*, *Isg15*, *Oas2* and *Ifit1* in *Pcgf3*^*+/+*^ and *Pcgf3*^*−/−*^ L929 cells treated with or without mouse IFN-α (50 ng/mL) for 6 h. **E** Immunoblot analysis of STAT1, STAT2, Viperin, IFIT1, PCGF3/5 and β-Actin in *Pcgf3*^*+/+*^ and *Pcgf3*^*−/−*^ L929 cells treated with or without mouse IFN-α (50 ng/mL) for 8 h. **F** qRT-PCR analysis mRNA of expression of *Mx1*, *Isg15*, *Oas2* and *Ifit1* in *Pcgf3*^*+/+*^ L929 cells transfected with the empty vector (*Pcgf3*^*+/+*^+Vector), and *Pcgf3*^*−/−*^ L929 cells transfected with the empty vector (*Pcgf3*^*−/−*^+Vector) and the PCGF3 overexpression vector (*Pcgf3*^*−/−*^+PCGF3) for 24 h, and then treated with or without mouse IFN-α (50 ng/mL) for 6 h. **G** Immunoblot analysis of STAT1, STAT2, Viperin, IFIT1, PCGF3/5 and β-Actin in *Pcgf3*^*+/+*^ L929 cells transfected with empty vector (*Pcgf3*^*+/+*^+Vector), and *Pcgf3*^*−/−*^ L929 cells transfected with empty vector (*Pcgf3*^*−/−*^+Vector) and Myc-PCGF3 vector (*Pcgf3*^*−/−*^+PCGF3) for 24 h, and then treated with or without mouse IFN-α (50 ng/mL) for 8 h. Every lane was normalized by its β-Actin. These values were shown below lanes (**E**, **G**) and quantitative results of three independent experiments were shown in Fig. S2D and S2E. Data are representative of three independent experiments (**E**, **G**) or shown as mean ± SD of *n* = 3 biological replicates (**A**–**D** and **F**), two-tailed unpaired Student’s t-test (**A**–**D** and **F**).

We then further examine how PCGF3 regulates antiviral responses. Knockdown of PCGF3 expression significantly increased the expression levels of ISGs in A549 cells upon IFN-α treatment compared with that in cells transfected with siRNA negative control (si-NC) (Fig. 2A). Silenced *Pcgf3* expression with si-*Pcgf3* also enhanced ISG expression in the mouse subcutaneous connective tissue L929 cells (Fig. S2A and Fig. 2B). Furthermore, the overexpression of PCGF3 in L929 cells weakened ISG expression after IFN-α treatment (Fig. 2C and Fig. S2B), indicating that PCGF3 inhibits ISG expression in both human and murine cells.

Furthermore, we generated Pcgf3-deficient (*Pcgf3*^*−/−*^) L929 cells using the CRISPR-Cas9 system, which did not express PCGF3 (Fig. S2C). Consistent with the effect of si-*Pcgf3* on ISG expression, knockout of *Pcgf3* increased the expression of ISG genes (*Isg15, Ifit1, Oas2* and *Mx1*) compared to *Pcgf3*^*+/+*^ L929 cells upon IFN-α treatment (Fig. 2D). The protein levels of ISGs (STAT1, STAT2, Viperin and IFIT1) also were increased in *Pcgf3*^*−/−*^ L929 cells compared to *Pcgf3*^*+/+*^ cells triggered by IFN-α (Fig. 2E and Fig. S2D). Conversely, the rescue of PCGF3 expression in *Pcgf3*^*−/−*^ cells significantly reduced these mRNA and protein levels of ISGs (Fig. 2F, G and Fig. S2E). Taken together, these results suggest that PCGF3 suppresses ISG expression at both the mRNA and protein levels in innate immune cells.

### The levels of nuclear PCGF3 remain steady upon IFN-I treatment

**Fig. 3:**
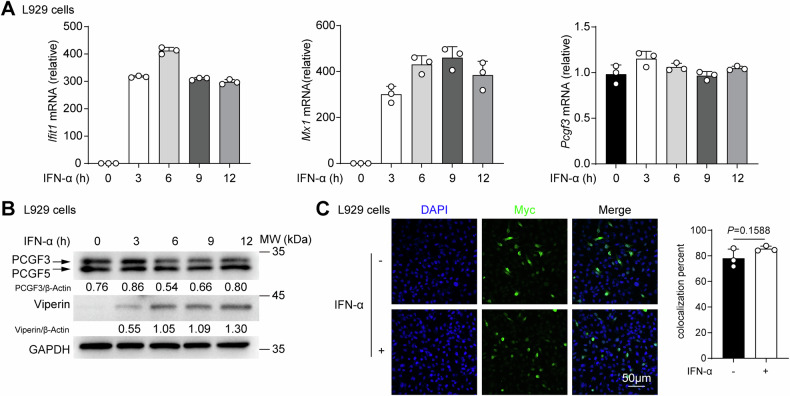
Nuclear PCGF3 levels remain stable after IFN-I treatment. **A** qRT-PCR analysis mRNA of expression of *Ifit1*, *Mx1* and *Pcgf3* in L929 cells treated with mouse IFN-α (50 ng/mL) for the indicated times. **B** Immunoblot analysis of Viperin, PCGF3/5 and GAPDH in L929 cells treated with mouse IFN-α (50 ng/mL) for the indicated times. **C** Immunofluorescence analysis and the colocalization quantitative results of Myc and DAPI of the Myc-tagged PCGF3 in *Pcgf3*^*−/−*^ L929 cells transfected with Myc-tagged PCGF3 vector treated with or without mouse IFN-α (50 ng/mL) for 6 h. Every lane was normalized by its GAPDH. These values were shown below lanes. Colocalization percent (Myc and DAPI) was measured by ImageJ. Data are representative of three independent experiments (**B**, **C**). Data are shown as mean ± SD of *n* = 3 biological replicates (1 field for each repeat experiments, **C**), two-tailed unpaired Student’s t-test (**C**).

We then investigated whether PCGF3 regulates ISG expression by modulating its own expression through IFN-I treatment. IFN-α did not change PCGF3 expression, but significantly increased ISGs (*Ifit1, Mx1* and *Viperin*) expression, either mRNA (Fig. 3A) or protein (Fig. 3B) in L929 cells. Immunofluorescence assays showed the nuclear localization of PCGF3 with or without IFN-α treatment (Fig. 3C). These results show that the expression level and location of PCGF3 do not change upon IFN-α treatment. Given the critical role of PCGF3 in PRC1, we speculate that PCGF3 may be involved in the transcription of ISGs in the nucleus.

### PCGF3 is recruited to ISRE and represses STAT1 to bind DNA

**Fig. 4:**
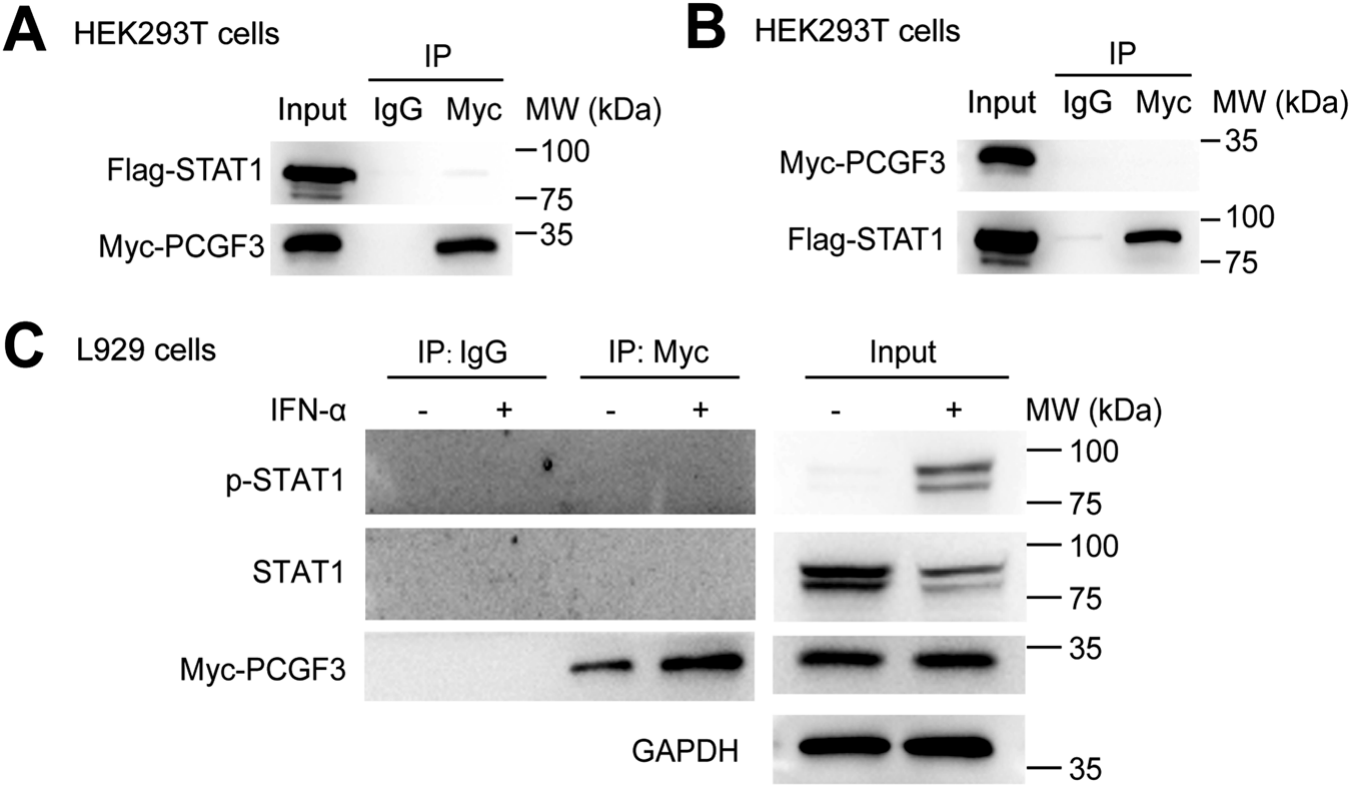
The inhibition of PCGF3 to ISG is independent of binding to STAT1. **A**, **B** Immunoprecipitation analysis of the interaction between STAT1 and PCGF3 in HEK293T cells cotransfected with Flag-STAT1, Myc-PCGF3. **C** Immunoprecipitation analysis of the interaction between STAT1/p-STAT1 and PCGF3 in *Pcgf3*^*−/−*^ L929 cells transfected with Myc-PCGF3 treated with or without mouse IFN-α (50 ng/mL) for 3 h. Data are representative of three independent experiments (**A**–**C**).

We then investigated the molecular mechanism by which PCGF3 inhibits IFN-I-mediated ISGs expression. The transcription factor STAT1 plays an important role in ISGs expression. Notably, PCGF3 was not associated with STAT1 in HEK293T cells co-expressing Myc-tagged PCGF3 and Flag-tagged STAT1 (Fig. 4A, B). Consistently, PCGF3 did not interact with either STAT1 or p-STAT1 in *Pcgf3*^*−/−*^ L929 cells overexpressing Myc-tagged PCGF3 with or without IFN-α treatment (Fig. 4C). These results suggest that there is no interaction between PCGF3 and STAT1 in the nucleus.

**Fig. 5:**
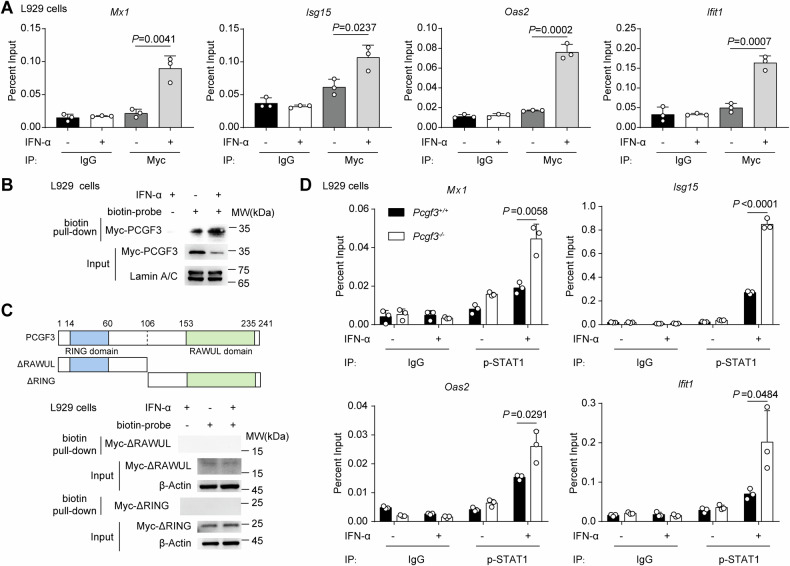
Nuclear PCGF3 is recruited to ISRE and represses STAT1 to bind DNA. **A** Chromatin immunoprecipitation analysis of PCGF3 binding in promoters of *Mx1*, *Isg15*, *Oas2* and *Ifit1* in *Pcgf3*^*−/−*^ L929 cells transfected with Myc-PCGF3 treated with or without mouse IFN-α (50 ng/mL) for 0.5 h. **B** DNA pull-down assay of PCGF3-overexpressed (Myc-PCGF3) *Pcgf3*^*−/−*^ L929 cells treated with or without mouse IFN-α (50 ng/mL) for 0.5 h. **C** The domains of mouse PCGF3 (top). DNA pull-down assay of truncation-overexpressed (ΔRAWUL and ΔRING, Myc-tagged) *Pcgf3*^*−/−*^ L929 cells treated with or without mouse IFN-α (50 ng/mL) for 0.5 h (bottom). **D** Chromatin immunoprecipitation analysis of p-STAT1 binding in promoters of *Mx1*, *Isg15*, *Oas2* and *Ifit1* in *Pcgf3*^*−/−*^ and *Pcgf3*^*+/+*^ L929 cells treated with or without mouse IFN-α (50 ng/mL) for 0.5 h. Data are representative of three independent experiments (**B**, **C**) or shown as mean ± SD of *n* = 3 biological replicates (**A**, **D**), two-tailed unpaired Student’s t-test (**A**, **D**).

It has been reported that PCGF3 is recruited to specific DNA sites as a transcriptional activator and drives the expression of many genes involved in mesoderm differentiation. We hypothesised that PCGF3 binds to IFN-stimulated response elements (ISREs) to regulate the transcription of ISGs. Using chromatin immunoprecipitation followed by qPCR analysis (ChIP-qPCR), we found that PCGF3 bound robustly to the promoter regulatory regions of the ISG genes in PCGF3-overexpressed *Pcgf3*^*−/−*^ L929 cells with IFN-α treatment (Fig. 5A and Fig. S3A). The DNA pull-down assay showed that more PCGF3 bound to biotinylated ISRE probes with IFN-α treatment compared to without treatment (Fig. 5B). We then determined the domain of PCGF3 that binds to ISRE sequences. PCGF3 contained two domains: RING domain (14–60 aa) and RAWUL domain (153-235 aa) [33], and DNA pull-down assay revealed that neither of these domains bound to ISRE (Fig. 5C), suggesting that both domains may be necessary for the recruitment of PCGF3 to ISRE.

We then investigated whether the recruitment of PCGF3 to ISRE prevents the binding of the interferon-stimulated gene factor 3 (ISGF3) complex (STAT1/STAT2/IRF9) to ISRE. ChIP-qPCR assay showed that p-STAT1 bound more strongly to the promoter regulatory regions of the ISG genes in *Pcgf3*^*−/−*^ L929 cells than in *Pcgf3*^*+/+*^ cells (Fig. 5D). Consistently, knockdown of *PCGF3* expression increased ISRE activity in THP-1 cells transfected with si-*PCGF3* (Fig. S3B, C). These results suggest that PCGF3 is recruited to ISRE and inhibits the binding of p-STAT1 to chromatin, resulting in the inhibition of ISG transcription.

### PCGF3 is reduced in DM patients

**Fig. 6:**
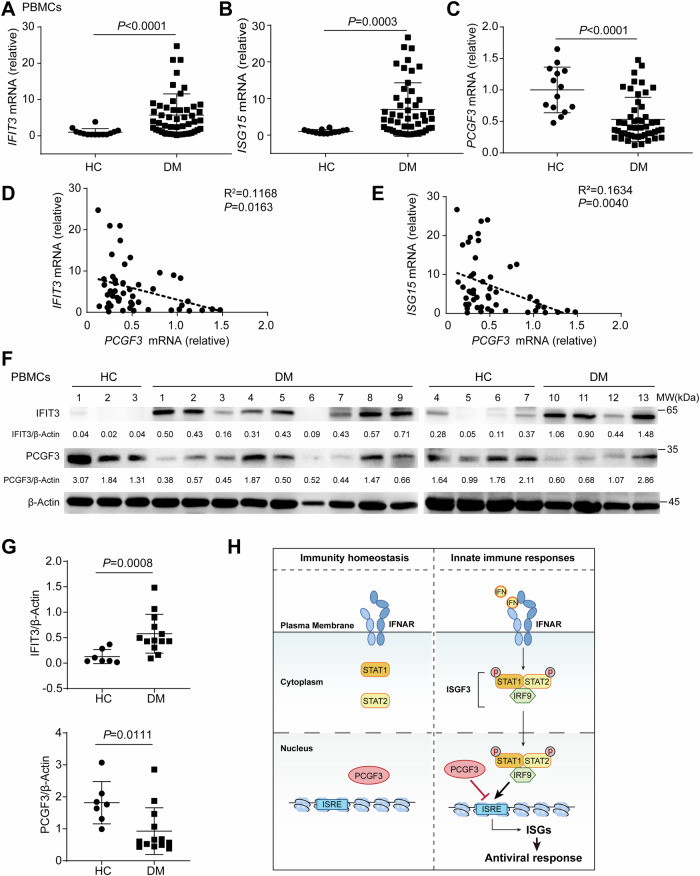
PCGF3 is reduced in DM patients. **A**–**C** qRT-PCR analysis mRNA of expression of *IFIT3* (**A**), *ISG15* (**B**) and *PCGF3* (**C**) in PBMCs from health controls (HC) and MDA5^+^ DM patients (DM). **D, E** Correlation analysis of the expression level of ISGs (*IFIT3* (**D**) and *ISG15* (**E**)) and *PCGF3* in PBMCs from MDA5^+^ DM patients. **F**, **G** Immunoblot analysis of IFIT3, PCGF3, and β-Actin in PBMCs from health controls (HC) and MDA5^+^ DM patients (DM). Every lane was normalized by its β-Actin. These values were shown below lanes. The quantitative results showed in (**G**). **H** Proposed working model that the recruitment of PCGF3 on ISRE prevents p-STAT1 binding to chromatin and inhibits ISG transcription. A Mann-Whitney test was used to analyze the statistical significance of data in (**A**–**C** and **G**). A Pearson correlation coefficient was used to evaluate the correlation of data in (**D**, **E**). Data are representative of three independent experiments (**F**).

Since PCGF3 down-regulates the expression of genes induced by IFN-I, we investigated the relationship between PCGF3 and ISGs in IFN-I-related autoimmune diseases. The key role of IFN-I in DM was supported by the identification of an IFN signature in muscle, blood and skin of DM patients [7–9]. We investigated the expression of ISGs in peripheral blood mononuclear cells (PBMCs) from 14 healthy controls (HC) and 49 patients with MDA5^+^ DM. As expected, the expressions of the ISGs (*IFIT3* and *ISG15*) were upregulated in MDA5^+^ DM patients (Fig. 6A, B), whereas PCGF3 expression was decreased in MDA5^+^ DM patients (Fig. 6C). Furthermore, the expression levels of PCGF3 were negatively correlated with either *IFIT3* or *ISG15* in MDA5^+^ DM patients (Fig. 6D, E). We investigated similar results for protein expression in MDA5^+^ DM patient samples (Fig. 6F, G). These results suggest that the reduction of PCGF3 expression enhances the expression of ISGs, which may be involved in the initiation and development of DM.

## Discussion

The IFN-I-IFNAR-STAT1-ISG signalling pathway is critical for antiviral immunity of the host, but prolonged activation of the signalling pathway is closely associated with several immune disorders. The mechanisms underlying the regulation of IFN-I signalling are not fully understood. Several RING domain-containing E3 ligases have been shown to ubiquitinate STAT1 and regulate STAT1-mediated transcriptional activity, thereby participating in the immune response [29]. However, it is unclear how other RING E3 ligases regulate the IFN-I-STAT1 signalling pathway and thus participate in the antiviral immune response and the pathogenesis of autoimmune diseases. In this study, we found for the first time that PCGF3, also known as RING finger protein 3 A (RNF3A), is a negative regulator of ISG transcription. Upon activation of the IFN-I pathway within a short time (0.5 h), PCGF3 could be recruited to ISRE, leading to reduced ISRE binding to p-STAT1 and repression of ISG transcription, suggesting that PCGF3 prevents p-STAT1 from binding to the ISRE promoter elements in ISGs. Besides, PCGF3 also can bind to RING1A, which may lead to the recruitment of PRC1 and continuous repression of ISG transcription (Fig. 6H).

The specific functions of PCGF3 have not been thoroughly investigated and little research has been conducted in the field of oncology. Previous studies have shown that PCGF3 mutations are key molecular alterations in intraductal papillary mucinous tumors [35], with PCGF3 being upregulated in pancreatic tumours and associated with signal transduction [36]. PCGF3 has also been reported to be a key component of the PRC1 complex, which mediates epigenetic histone modifications [32]. Specifically, the PCGF3/RING1B heterodimer presents a scaffold for PRC1 to assemble, and PCGF3 capable of influencing the catalytic activity of RING1B [33]. Moreover, PCGF3 is capable of binding USF1/2, leading to the recruitment of PRC1 to the USF1 binding [22]. The binding of STAT1 to the CIITA-GAS site is firmly stabilized by USF-1 [23]. We then speculated that USF-1 might facilitate the binding of STAT1 and PCGF3 to the promoters of interferon-stimulated genes (ISGs). Surprisingly, our results indicate that PCGF3 does not bind to STAT1. Therefore, how PCGF3 specifically binds to ISRE, whether it is dependent on USF1 or other proteins, remains to be investigated. Furthermore, the sites to which PRC1.3 can be recruited and the mechanisms of site-specific recruitment under specific circumstances remain incompletely understood. Here we report that PCGF3 is recruited to ISRE in an IFN-I-dependent manner and that this process depends on both domains of PCGF3. However, the specific mechanism by which PCGF3 inhibits the binding of the ISGF3 complex to the ISRE site remains unclear. Is there competition between PCGF3 and ISGF3 for binding to the ISRE? Furthermore, do structural or post-translational modification (PTM) changes in the PCGF3 and ISGF3 complexes influence their binding to the ISRE? These issues are indeed worthy of in-depth study in the future.

DM is a type I interferonopathy with the enhanced expression of ISGs as a major characteristic that distinguishes it from other myopathies [8]. In DM, ISGs are significantly upregulated in both inflamed muscle and PBMCs after pDCs infiltrate the tissue and secrete abnormal levels of IFN-I [10]. Serum IFN-α levels are associated with serum muscle enzymes in untreated DM patients and inversely associated with the duration of untreated disease [11]. Moreover, the higher levels of anti-Jo1 and anti-Ro (SSA) antibodies are associated with higher expression of ISGs on PBMCs and greater disease activity among patients with DM [37]. A prominent IFN-I signature was identified in the skin, muscle and blood of adults and patients with DM [7–9]. The IFN signature also appears to be correlated with disease activity in adult DM [38]. Anti-MDA5 antibody positive (MDA5^+^) patients seem to have a stronger IFN-I signature in blood and skin compared with MDA5^-^ patients [39, 40]. As MDA5^+^ patients have relatively mild muscle involvement and worse skin and lung disease compared to MDA5^-^ patients [41], it is feasible that the differences in IFN-I signatures are related to the degree of organ involvement. Here we observed that the level of PCGF3 mRNA was lower in PBMCs of MDA5^+^ DM patients compared to HC. In contrast, STAT1/2 were highly enriched in the peripheral B and T cells of MDA5^+^ patients. This suggests that less PCGF3 and more STAT1 may be recruited to ISRE in DM patients compared to HC, leading to high expression of ISGs [42]. Given the inverse correlation between the expression levels of ISGs and PCGF3 in PBMCs of DM patients, PCGF3 expression may serve as a biomarker of disease activity in DM.

In conclusion, our findings suggest that nuclear PCGF3 plays an important role in the IFN-STAT1-ISG signalling. Further investigation is required to fully understand the epigenetic regulatory functions of PCGF3 in the regulation of immune pathways in response to various stimuli. In addition, in vivo studies, clinical trials, and larger statistical analyses will be required to validate of PCGF3 as a prognostic biomarker for autoimmune diseases.

## Materials and methods

### Cells

A549, L929, HEK293T and RAW264.7 cell lines were obtained from ATCC. *Pcgf3* deficient L929 cells were generated using CRISPR-Cas9 system with guide RNA (5′-GAGCTGCCTGGTGAAGTATC-3′) targeting exon 2 of Pcgf3. Briefly, the PX458 plasmid (Addgene, 48138), which had been inserted the guide RNA, was transfected into L929 cells. Flow cytometry was used to select EGFP-positive cells as single clones. Single clones were identified by Western Blot. A549 cells were cultivated in F12K medium (Boster, PYG0036) with 10% FBS (Gibco, 10099-141 C). L929, HEK293T and RAW264.7 cells were cultivated in DMEM medium (Corning, 10-013-CV) with 10% FBS. NF-κB-SEAP and IRF-Lucia luciferase reporter monocyte cell line (THP1-Dual™ cells, thpd-nfis) was from InvivoGen. THP1-Dual™ cells were cultivated according to the instructions of the manufacturer. Peritoneal macrophages were harvested from mice 4 days after thioglycollate (Sigma, T0632) medium injection.

### Healthy controls and DM patients

Healthy controls (HC) had no history of autoimmune diseases. MDA5^+^ DM patients with concurrent infection were excluded from this study. All MDA5^+^ DM patients fulfilled the American College of Rheumatology (ACR) classification criteria for MDA5^+^ DM. This study was approved by the Research Ethics Board of the Affiliated Hospital of Inner Mongolia Medical University Donors had been informed the usage of samples, and written informed consent was signed before sample collection. Information of HC and MDA5^+^ DM patients were listed in Supplementary Table S3.

The peripheral blood mononuclear cells (PBMCs) from HC and MDA5^+^ DM patients were separated by Ficoll-Hypaque density gradients centrifugation. Lymphocyte separation medium (Solarbio, P8610) was used to separate PBMCs following the manufacturer’s instructions, in which the erythrocytes were removed by lysing buffer (Solarbio, R1010).

### Reagents, antibodies and plasmids

Recombinant human IFN-α(2a) (300-02AA, 10 ng/ml for cell treatment) was from PeproTech. Recombinant mouse IFN-α2 (CK83, 50 ng/ml for cell treatment) was from Phorbol 12-myristate 13-acetate (PMA, HY-18739) and 4′,6-diamidino-2-phenylindole dilactate (DAPI, HY-D1738) were from MedChemExpress. Anti-PCGF3 + PCGF5 antibody (ab201510) and anti-Viperin antibody (ab107359) was from Abcam. Anti-RNF3 (PCGF3) antibody (ARG63252) was from Arigo. IFIT1 polyclonal antibody (abs 149003) was from Absin. IFIT3 polyclonal antibody (15201-1-AP) was from Proteintech. Antibodies specific for Myc-tag (2276), DYKDDDDK-tag (Flag-tag, 14793), STAT1 (14994), p-STAT1 (Tyr701, 9167), STAT2 (72604), β-Tubulin (2146), Lamin A/C (2032) and normal rabbit IgG (2729) were from Cell Signaling Technology. Normal mouse IgG (A7050) was from Beyotime. Anti-GAPDH antibody (M171-3) and Anti-β-Actin antibody (M177-3) were from MBL. HRP-tagged anti-ribbat-IgG antibody (ZB-2301) and HRP-tagged anti-mouse-IgG antibody (ZB-2305) were from ZSGB-Bio. QUANTI-Luc™ (rep-qlc1) was from InvivoGen. The mouse Pcgf3, Stat1 and Ring1a ORF sequence were amplified from L929 cells cDNA and inserted into pcDNA3.1 vectors.

### Virus

GFP-VSV and VSV were propagated and amplified by infection of a monolayer of Vero cells. After 48 h of infection, the supernatant was harvested and clarified by centrifugation. Viral titer was determined by TCID_50_ on 293 T cells. Briefly, 293 T cells in 96-well cell culture plates were infected by virus with different dilution ratios for 48 h. TCID_50_ was calculated by Reed-Muench method.

### Cell transfection

For RNA interference, siRNAs were transfected at the concentration of 50 nM using Lipofectamine RNAiMAX (Thermo Scientific, 13778100) according to the instructions of the manufacturer. Supplementary Table S1 listed all siRNAs had been used in this study. All plasmids were transfected at the concentration of 1 μg/mL using Lipofectamine 3000 (Thermo Scientific, L3000150) according to the instructions of the manufacturer.

### Quantitative reverse transcription PCR (qRT-PCR)

RNAfast2000 Total RNA Extraction Kit (Fastgene, 220011) was used extract total RNAs from cells according to the instructions of the manufacturer. Then 2 μg of RNAs was reversely transcribed to cDNA with ReverTra Ace qPCR RT Master Mix (Toyobo, FSQ-201). Diluted cDNA was used to quantify the relative level of mRNA with SYBR Green Realtime PCR Master Mix (Toyobo, QPK201) by Biorad CFX96 in 96-well plate. The expression of *GAPDH* and *Gapdh* were used to normalize Cycle threshold (CT) of every sample. Primers for qRT-PCR are listed in Supplementary Table S2.

### Co-immunoprecipitation assay

Cells were collected with pre-cold PBS and lysed with IP lysis buffer (Thermo Scientific, 87787) containing protease inhibitors (Beyotime, P1005) for 30 min on ice. The supernatant was collected after centrifugation, and a part of it was reserved as Input. Lysate with 100 μg of total proteins was incubated with the specific antibody or the same amount of normal IgG overnight. The amount of antibody for immunoprecipitation was determined by the instructions of the manufacturer. Then 10 μL of protein G dynabeads (Thermo Scientific, 88847) were added into mixture and incubated for another two hours. The dynabeads were washed with IP lysis buffer and separated by MagnaRack Magnetic Separation Rack for five times. 10 μL of 50 mM NaOH was used to dissociate proteins was from dynabeads, and the supernatant was separated by MagnaRack Magnetic Separation Rack. After neutralizing by 5 μL of 1 M Tris-HCl, the supernatant was ready for immunoblot assay.

### Western blot assay

Cells were collected with pre-cold PBS and lysed with RIPA buffer (Huaxingbio) containing with protease inhibitor cocktail (Beyotime, P1005) and phosphatase inhibitor cocktail (Sangon, C500017). Protein concentration of lysate was determined by the BCA Protein Assay Kit (Beyotime, P0011) according to the manufacturers’ instructions. Cell lysates were boiled for 10 min at 95°C with Protein Loading Buffer (Transgen, DL101), then loaded into 10% SDS-PAGE. Proteins were transferred to PVDF membranes (1620177, Bio-Rad). The membranes were blocked and then incubated with certain primary antibodies overnight at 4°C. The membranes were washed and incubated with anti-rabbit or -mouse IgG-HRP for 1 h. Protein bands were visualized with the western blotting detection system Tanon-5200 (Bio-Tanon, China). Gray value analysis was done by ImageJ (v.l.50 g, NIH) software.

### Immunofluorescence assay

Cells were seeded on sterile coverslip coated with poly-L-Lysine and placed into the 24-well cell culture plate. Following treatment with or without IFN-α, cells were fixed with 4% paraformaldehyde at room temperature. Then cells were permeabilized with 0.2% Triton X-100 in PBS and were blocked with 1% BSA. Myc-tag was stained with anti-Myc antibody (1:300 dilution) overnight. After that, cells were stained by Alexa Fluor 488 secondary antibody (Thermo Scientific, A32731, 1:500) for 1 hour. Between every step, cells were washed by 1×PBS for three times. After stained by DAPI and fixed, cells were observed with a Leica TCS SP8 gSTED 3×with 63×/1.4 oil objective lens.

### Chromatin immunoprecipitation (ChIP)

SimpleChIP Enzymatic Chromatin IP Kit (CST, 9003) was used for ChIP-qPCR assays according to the instructions of the manufacturer. Briefly, chromatin was cross-linked by 1% formaldehyde. After nuclei preparation, chromatin was digested to a length of approximately 150–900 bp, which was ready for immunoprecipitation. Anti-Myc-tag antibody (1:250 dilution) and normal mouse IgG (1:100 dilution) were used for immunoprecipitation. After immunoprecipitation, DNA was purified for qPCR analysis. The locations of primers showed in Fig. S3A, and the sequences of primers were listed in Supplementary Table S1.

### Luciferase reporter assay

THP1-Dual™ cells were induced to differentiate by PMA for luciferase reporter assay according to the instructions of the manufacturer. After the transfection of siRNA targeting PCGF3, THP1-Dual™ cells were treated with IFN-α for 24 hours. In total 10 μL of supernatant was collected for measurements of luciferase activity. A total of 50 μL of QUANTI-Luc™ was added into supernatant. The luminometer was set with the parameters: end-point measurement with a 0.1 second reading time.

### DNA pull-down assay

Cells were collected with pre-cold PBS. For PCGF3 overexpressed *Pcgf3*^*−/−*^ L929 cells, soluble nuclear proteins were extracted by NE-PER Nuclear and Cytoplasmic Extraction Reagents (Thermo Scientific, 78835) according to the instructions of the manufacturer. PCGF3 (1–106aa) and PCGF3 (107–241aa) overexpressed *Pcgf3*^*−/−*^ L929 cells were lysed with IP lysis buffer (Thermo Scientific, 87787) containing protease inhibitors (Beyotime, P1005). Biotin labeled and unlabeled DNA probes (5′- TTTCAGTTTCAGTTTCAGTTTCAGTTTCAGTTTC-3′) contained three canonical ISRE motif (5′-TTTCNNTTTC-3′). 2.5 ug of DNA probes was added into 400 μg of total proteins and incubated overnight. Then 15 μL of Streptavidin C1 dynabeads (Thermo Scientific, 65001) were added into mixture and incubated for another 1 h. The dynabeads were washed with IP lysis buffer and separated by MagnaRack Magnetic Separation Rack for five times. Then the dynabeads were boiled in protein loading buffer and ready for immunoblot assay.

### Statistical analysis

The analysis methods are shown in figure legends. All data was analyzed with GraphPad Prism 8 or SPSS version 19. P values were shown on each figure. P values less than 0.05 were regarded as statistically significant.

## Supplementary information

Supplemental material

Original Data

## Data Availability

The datasets used and/or analyzed during the current study are available from the corresponding author upon reasonable request.
