# Supplementary material for: Nuclear PCGF3 inhibits the antiviral immune response by suppressing the interferon-stimulated gene
Source: Cell Death Discov. 2024 Oct 5;10:429. doi: 10.1038/s41420-024-02194-x (PMC11455894; doi:10.1038/s41420-024-02194-x)
Supplement: Supplementary file 1 — Supplemental material [file 41420_2024_2194_MOESM1_ESM.docx]

**Supplementary Figures**


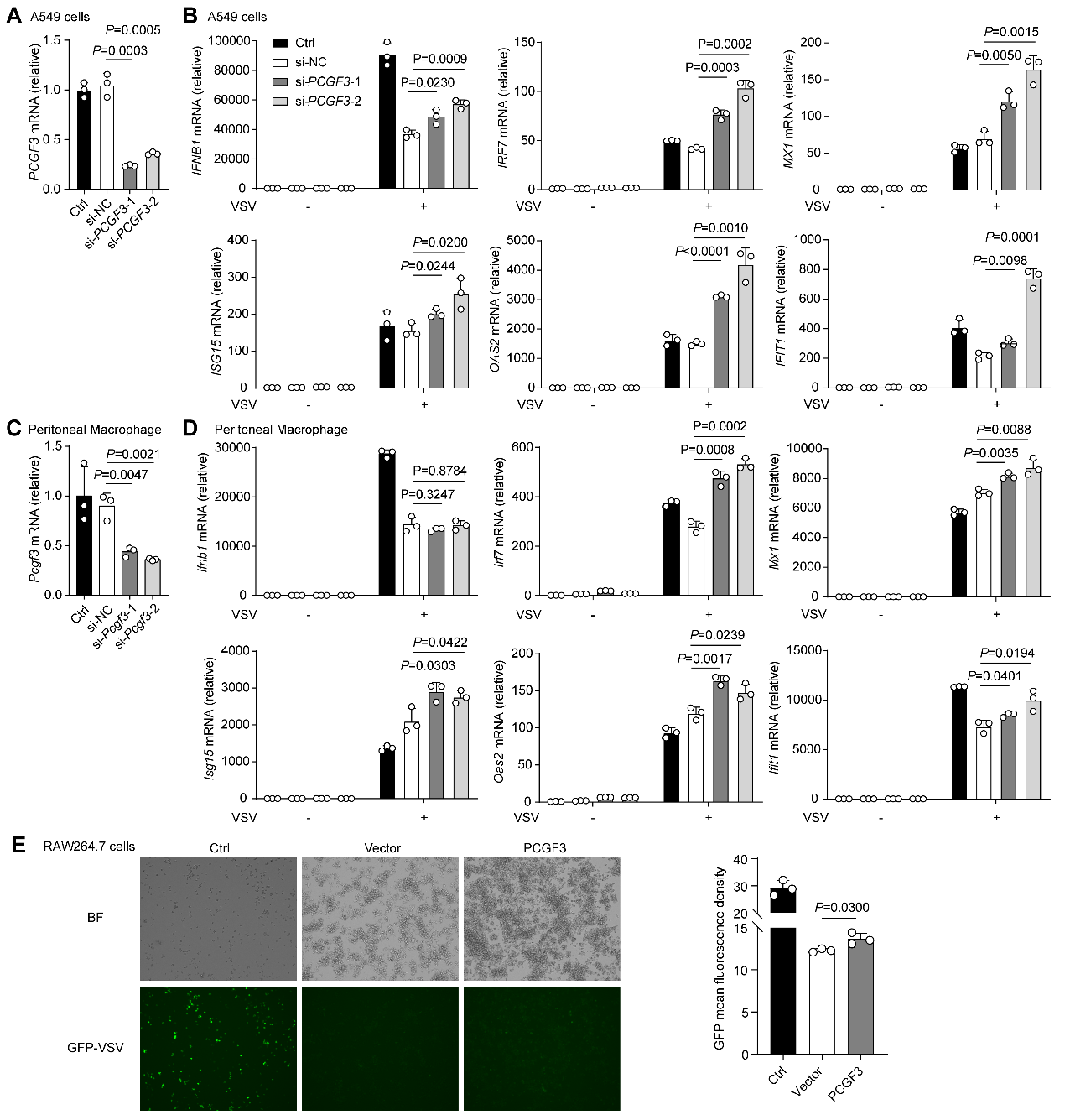


**Figure S1. PCGF3 inhibits antiviral response in cells**

**A,** qRT-PCR analysis mRNA of expression of *PCGF3* in A549 cells transfected with si-*PCGF3* or si-NC for 48 h. Cells without treatment were Ctrl.

**B,** qRT-PCR analysis mRNA of expression of *IFNB1*, *IRF7*, *MX1*, *ISG15*, *OAS2* and *IFIT1* in A549 cells without treatment (Ctrl) and A549 cells transfected with si-*PCGF3* (*si-PCGF3*-1 and *si-PCGF3*-2) or si-NC for 48 h, and then infected with VSV (MOI=1) for 8 h.

**C,** qRT-PCR analysis mRNA of expression of *Pcgf3* in mouse peritoneal macrophages cells transfected with si-*Pcgf3* or si-NC for 48 h. Cells without treatment were Ctrl.

**D,** qRT-PCR analysis mRNA of expression of *Ifnb1*, *Irf7,* *Mx1*, *Isg15*, *Oas2* and *Ifit1* in mouse peritoneal macrophages without treatment (Ctrl) and mouse peritoneal macrophages transfected with si-*Pcgf3* (*si-Pcgf3*-1 and *si-Pcgf3*-2) or si-NC for 48 h, and then infected with VSV (MOI=1) for 8 h.

**E,** Typical images of 3 biological replicates of RAW264.7 cells without treatment (Ctrl) and RAW264.7 cells transfected with vector (Vector) or PCGF3 plasmids (PCGF3) for 24 h, and then infected with GFP–VSV (MOI=1) for 8 h (left). BF: Bright Field. The GFP mean fluorescence intensity of 3 biological replicates (right).

Images in (E) was captured by EVOS system (Thermofisher). Mean fluorescence intensity was measured by ImageJ. Data are shown as mean ± SD of n = 3 biological replicates (1 field for each repeat experiments, E), two-tailed unpaired Student's t-test (A-E).


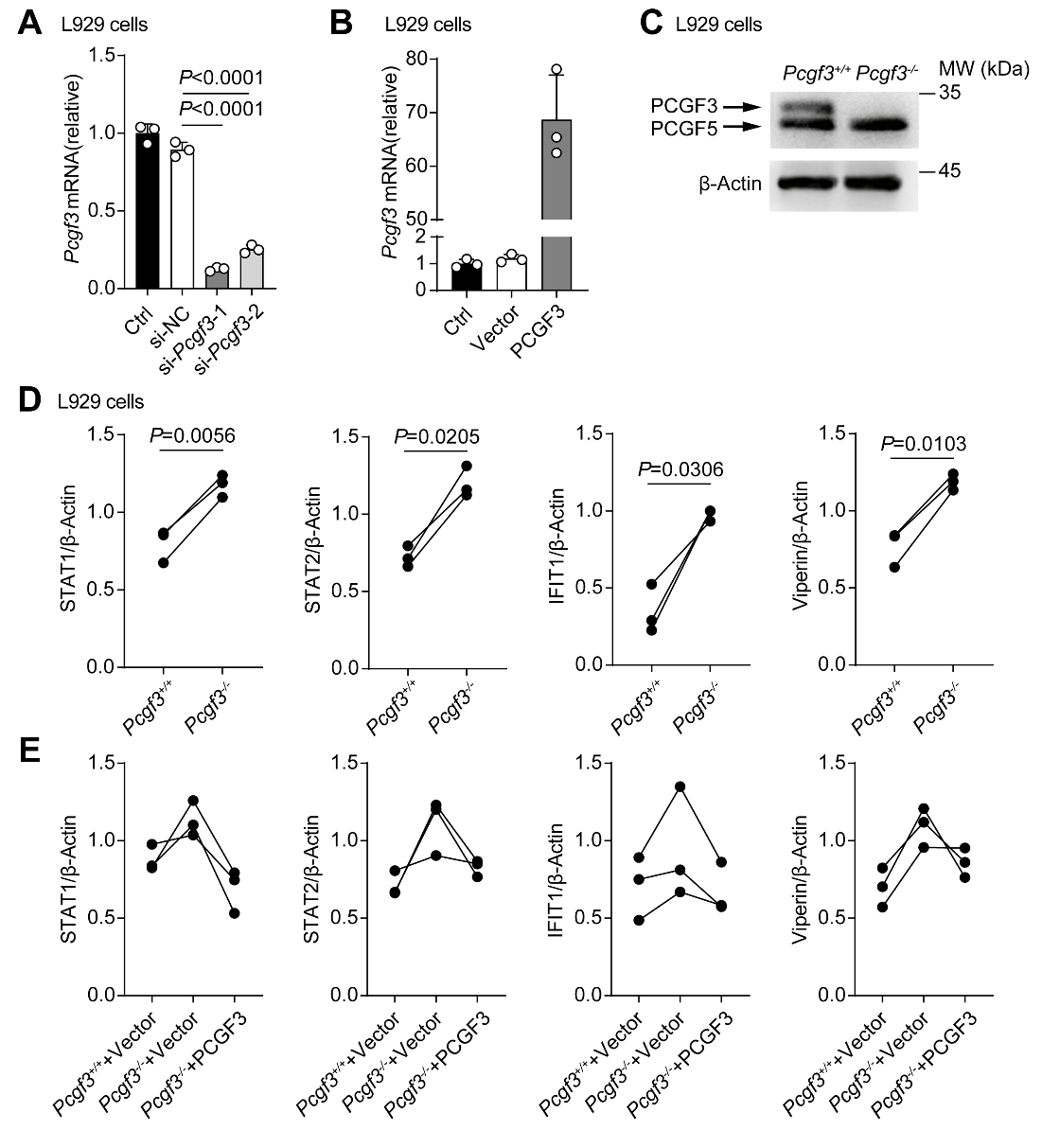


**Figure S2. PCGF3 inhibits the expression of ISGs.**

**A,** qRT-PCR analysis mRNA of expression of *Pcgf3* in L929 cells transfected with si-*Pcgf3* or si-NC for 48 h. Cells without treatment were Ctrl.

**B,** qRT-PCR analysis mRNA of expression of *Pcgf3* in L929 cells without treatment (Ctrl) and L929 cells transfected with the empty vector (Vector) or the PCGF3 overexpression vector (PCGF3) for 24 h.

**C,** Immunoblot analysis of PCGF3/5 and β-Actin in *Pcgf3^+/+^* and *Pcgf3^-/-^* L929 cells.

**D,** The quantitative results of Fig. 2E (after IFN-α treatment, lane 2 and 4). Line charts showed the results from three replications.

**E,** The quantitative results of Fig. 2G (after IFN-α treatment, lane 2, 4 and 6). Line charts showed the results from three replications.

Data are representative of three independent experiments (C) or shown as mean ± SD of n = 3 biological replicates (A, B, D and E), two-tailed unpaired Student's t-test (A, B, D and E).


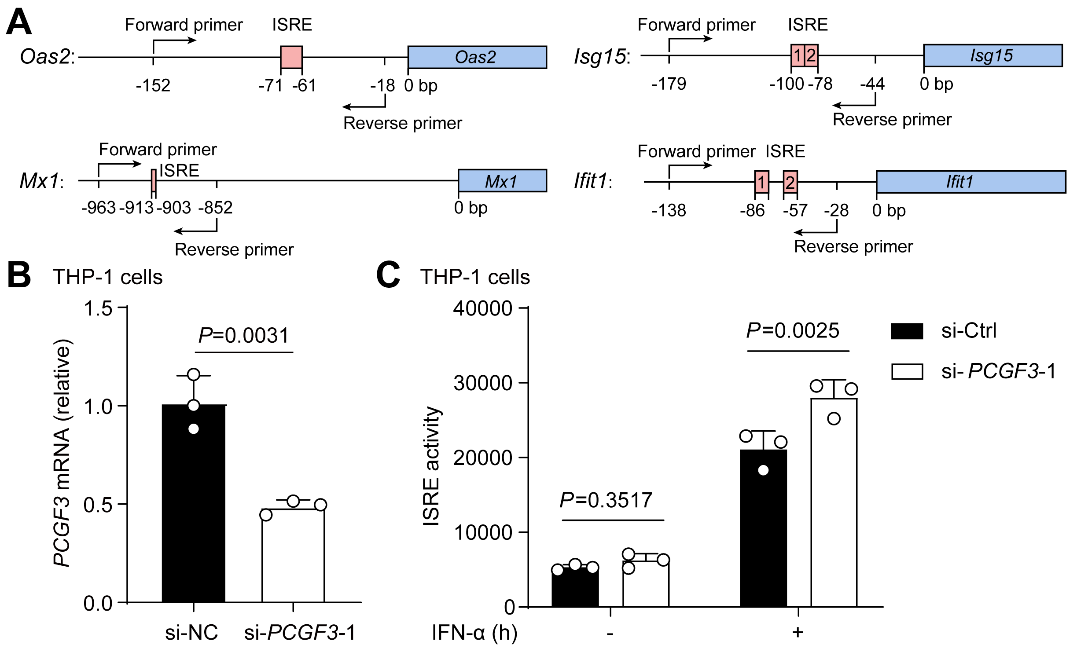


**Figure S3. Nuclear PCGF3 is recruited to ISRE and represses STAT1 to bind DNA**

**A,** The location of canonical ISRE motif (5’-TTTCNNTTTC-3’) and primers of ChIP-qPCR.

**B,** qRT-PCR analysis mRNA of expression of *PCGF3* in THP1-Dual™ cells transfected with si-*PCGF3* or si-NC for 48 h.

**C,** Luciferase reporter assay of ISRE activity in THP1-Dual™ cells transfected with si-NC or si-*PCGF3* for 48 h, and then treated with or without human IFN-α (10 ng/mL) for 12 h.

Data are shown as mean ± SD of n = 3 biological replicates (B, C), two-tailed unpaired Student's t-test (B, C).

**Supplementary Table S1**

**Primers used for quantitative PCR in this study**

| **Targets** | **Forward primer** | | **Reverse primer** |
| --- | --- | --- | --- |
| *GAPDH* | GGAGCGAGATCCCTCCAAAAT | GGCTGTTGTCATACTTCTCATGG | |
| *PCGF3* | AGGTGCCGGGAGACATCAA | GAACTGTCGTCTGCTTTGGTT | |
| *IFIT1* | GCGCTGGGTATGCGATCTC | CAGCCTGCCTTAGGGGAAG | |
| *IFIT3* | AACTACGCCTGGGTCTACTATCACTT | GCCCTTTCATTTCTTCCACAC | |
| *ISG15* | GGACAAATGCGACGAACC | CCCGCTCACTTGCTGCTT | |
| *OAS2* | CTCAGAAGCTGGGTTGGTTTAT | ACCATCTCGTCGATCAGTGTC | |
| *MX1* | GTTTCCGAAGTGGACATCGCA | CTGCACAGGTTGTTCTCAGC | |
| *IFNB1* | GTCAGAGTGGAAATCCTAAG | ACAGCATCTGCTGGTTGAAG | |
| *IRF7* | GCTGGACGTGACCATCATGTA | GGGCCGTATAGGAACGTGC | |
| *Gapdh* | AGGTCGGTGTGAACGGATTTG | TGTAGACCATGTAGTTGAGGTCA | |
| *Pcgf3* | TGCAGCGGCTACCTCATTG | CCGATATACTGTAATGGGTGGCT | |
| *Ifit1* | CTGAGATGTCACTTCACATGGAA | GTGCATCCCCAATGGGTTCT | |
| *Isg15* | GGTGTCCGTGACTAACTCCAT | TGGAAAGGGTAAGACCGTCCT | |
| *Oas2* | TTGAAGAGGAATACATGCGGAAG | GGGTCTGCATTACTGGCACTT | |
| *Mx1* | GACCATAGGGGTCTTGACCAA | AGACTTGCTCTTTCTGAAAAGCC | |
| *Ifnb1* | ATGAGTGGTGGTTGCAGGC | TGACCTTTCAAATGCAGTAGATTCA | |
| *Irf7* | GAGACTGGCTATTGGGGGAG | GACCGAAATGCTTCCAGGG | |
| *Ifit1* promoter | AAACCCCACAGTGCCCCACAGT | GGGGGTGTGCTCTTTTCAGTCAG | |
| *Isg15* promoter | GAGCCAGTCCCTTTCCTTCC | GTGAAGAGGCGGAGTTTCCA | |
| *Oas2* promoter | AAGCTGGGGTGTCCTTGGGAG | GTCCAAAGCCCCACCCCTAAC | |
| *Mx1* promoter | CTATTTCTCAGCATAACAC | CATCCTCTTCTTCCACTCCC | |
| VSV | ACGGCGTACTTCCAGATGG | CTCGGTTCAAGATCCAGGT | |

**Supplementary Table S2**

**List of the siRNA target sequence**

| **Name** | **Target sequence** | **Manufacturer** | |
| --- | --- | --- | --- |
| *PCGF3* siRNA1 (si-*PCGF3*-1) | Assay ID s20222 | | Thermo Fisher Scientific |
| *PCGF3* siRNA2 (si-*PCGF3*-2) | Assay ID s20223 | | Thermo Fisher Scientific |
| *Pcgf3* siRNA1 (si-*Pcgf3*-1) | GGGAATTCTATCACAAACT | | Ribobio |
| *Pcgf3* siRNA2 (si-*Pcgf3*-2) | CCAAAGCAGACGACAATTC | | Ribobio |
| si-NC | siN0000002-1-5 | | Ribobio |

**Supplementary Table S3**

**Information of healthy controls (HC) and MDA5^+^ DM patients (DM)**

|  | **Gender (F for female and M for male)** | **Age** |
| --- | --- | --- |
| HC1 | F | 55 |
| HC2 | F | 60 |
| HC3 | F | 58 |
| HC4 | F | 50 |
| HC5 | M | 48 |
| HC6 | M | 37 |
| HC7 | F | 34 |
| HC8 | F | 34 |
| HC9 | F | 48 |
| HC10 | M | 31 |
| HC11 | M | 46 |
| HC12 | F | 31 |
| HC13 | F | 30 |
| HC14 | F | 31 |
| DM1 | M | 48 |
| DM2 | F | 51 |
| DM3 | F | 53 |
| DM4 | M | 43 |
| DM5 | F | 75 |
| DM6 | F | 40 |
| DM7 | F | 52 |
| DM8 | F | 32 |
| DM9 | F | 55 |
| DM10 | M | 44 |
| DM11 | F | 48 |
| DM12 | F | 57 |
| DM13 | F | 69 |
| DM14 | F | 47 |
| DM15 | M | 62 |
| DM16 | F | 55 |
| DM17 | F | 32 |
| DM18 | F | 44 |
| DM19 | F | 45 |
| DM20 | F | 51 |
| DM21 | F | 53 |
| DM22 | M | 60 |
| DM23 | F | 48 |
| DM24 | M | 31 |
| DM25 | M | 31 |
| DM26 | M | 38 |
| DM27 | F | 50 |
| DM28 | M | 56 |
| DM29 | F | 58 |
| DM30 | F | 45 |
| DM31 | F | 46 |
| DM32 | M | 43 |
| DM33 | F | 21 |
| DM34 | M | 28 |
| DM35 | M | 67 |
| DM36 | M | 15 |
| DM37 | M | 54 |
| DM38 | F | 62 |
| DM39 | F | 61 |
| DM40 | M | 58 |
| DM41 | F | 76 |
| DM42 | F | 34 |
| DM43 | F | 41 |
| DM44 | F | 59 |
| DM45 | F | 43 |
| DM46 | M | 45 |
| DM47 | F | 63 |
| DM48 | M | 42 |
| DM49 | F | 56 |
