# Supplementary material for: Nuclear PCGF3 inhibits the antiviral immune response by suppressing the interferon-stimulated gene
Source: Cell Death Discov. 2024 Oct 5;10:429. doi: 10.1038/s41420-024-02194-x (PMC11455894; doi:10.1038/s41420-024-02194-x)

Figure 2E

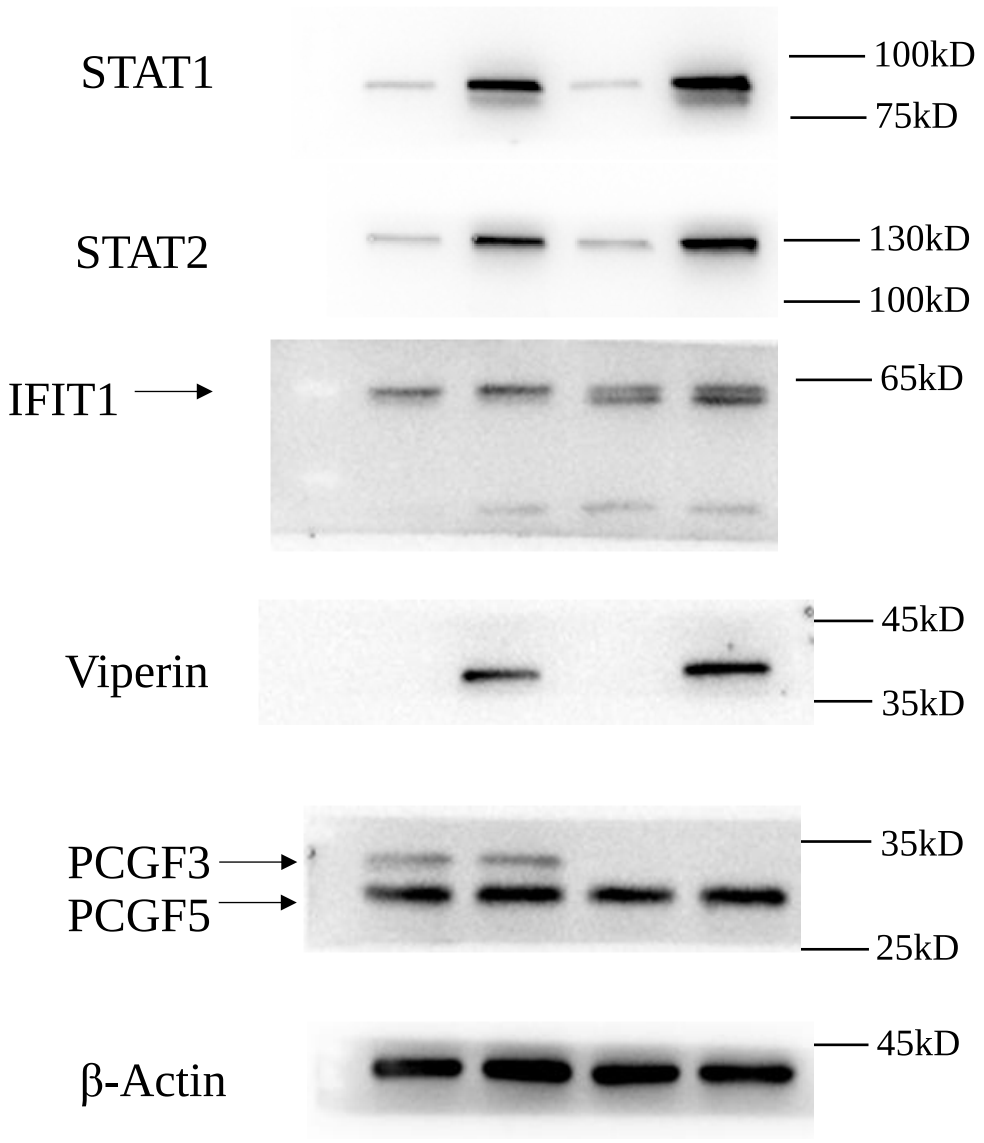
STAT1
100kD
75kD
STAT2
130kD
100kD
IFIT1
65kD
Viperin
45kD
35kD
PCGF3
PCGF5
35kD
25kD
β-Actin
45kD

Figure 2G

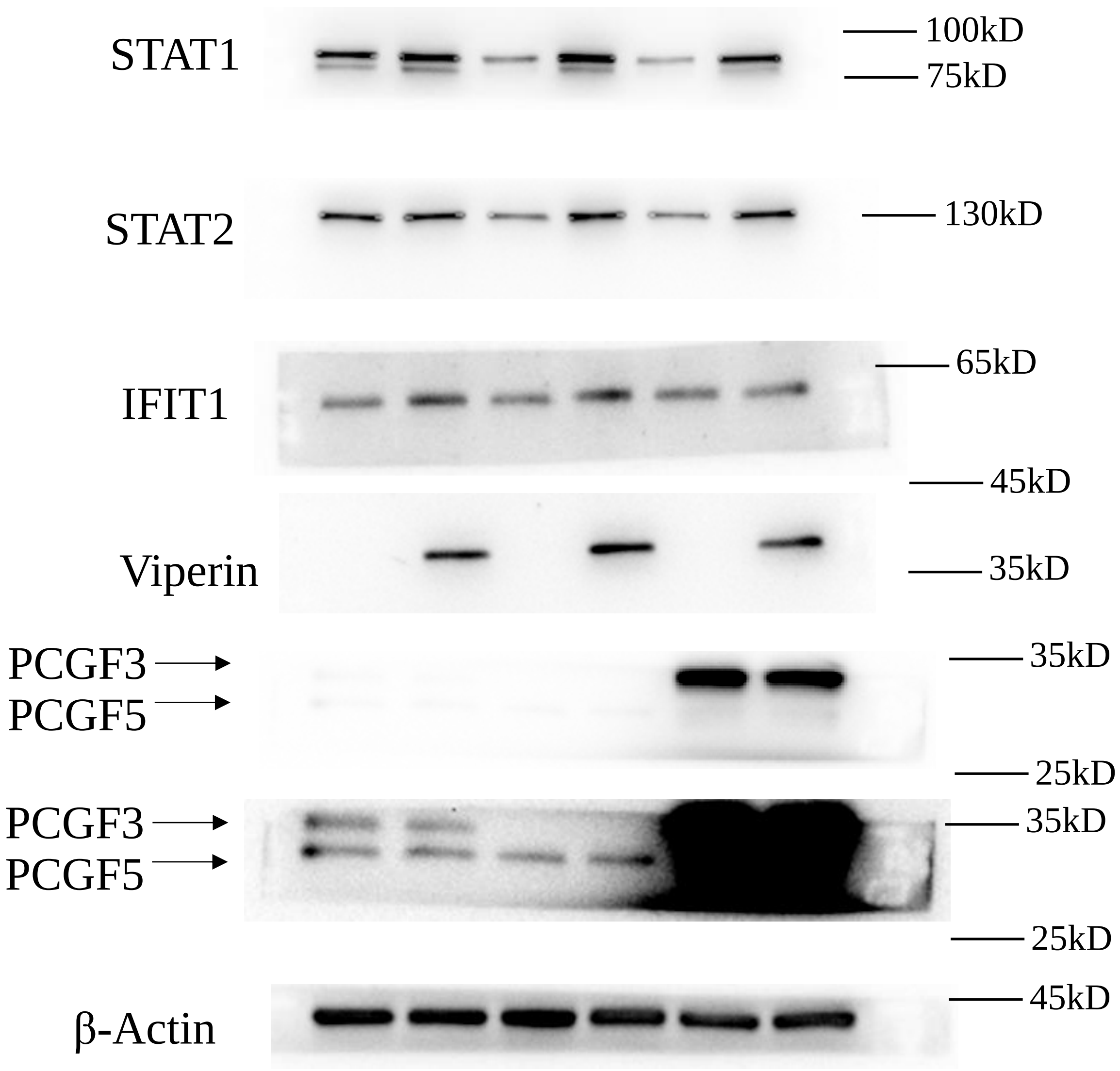
STAT1
100kD
75kD
STAT2
130kD
IFIT1
65kD
45kD
Viperin
35kD
PCGF3
PCGF5
35kD
25kD
PCGF3
PCGF5
35kD
25kD
β-Actin
45kD

Figure 3B

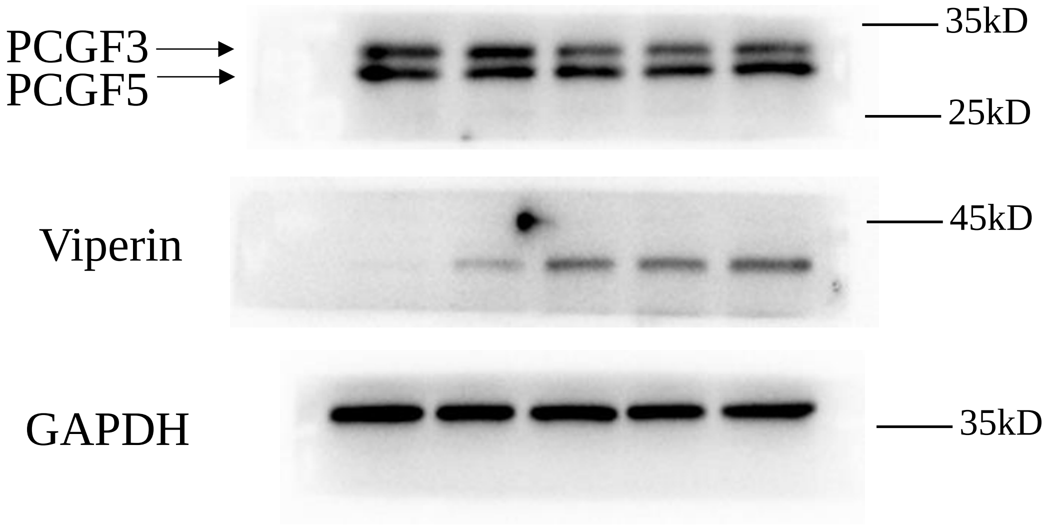

Figure 4A

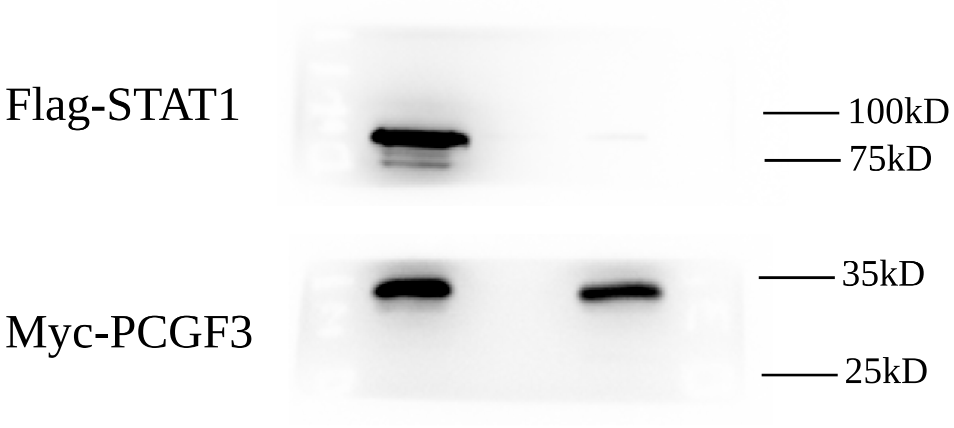

Figure 4B

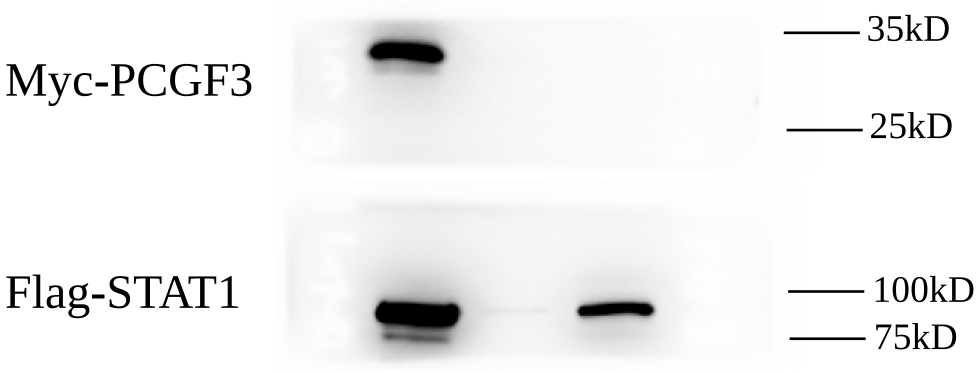

Figure 4C

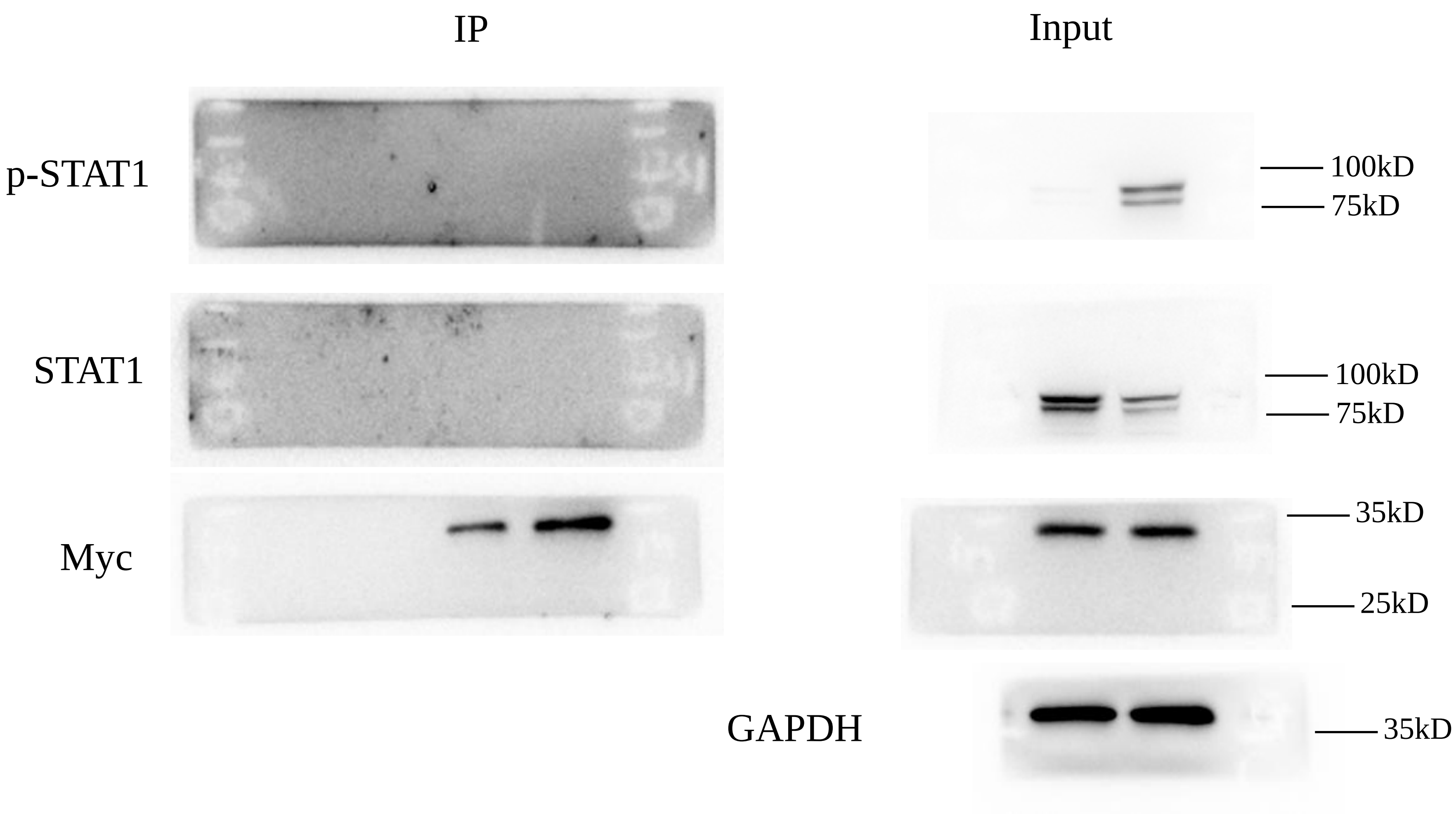
IP
Input
p-STAT1
STAT1
Myc
GAPDH
100kD
75kD
100kD
75kD
35kD
25kD
35kD

Figure 5B

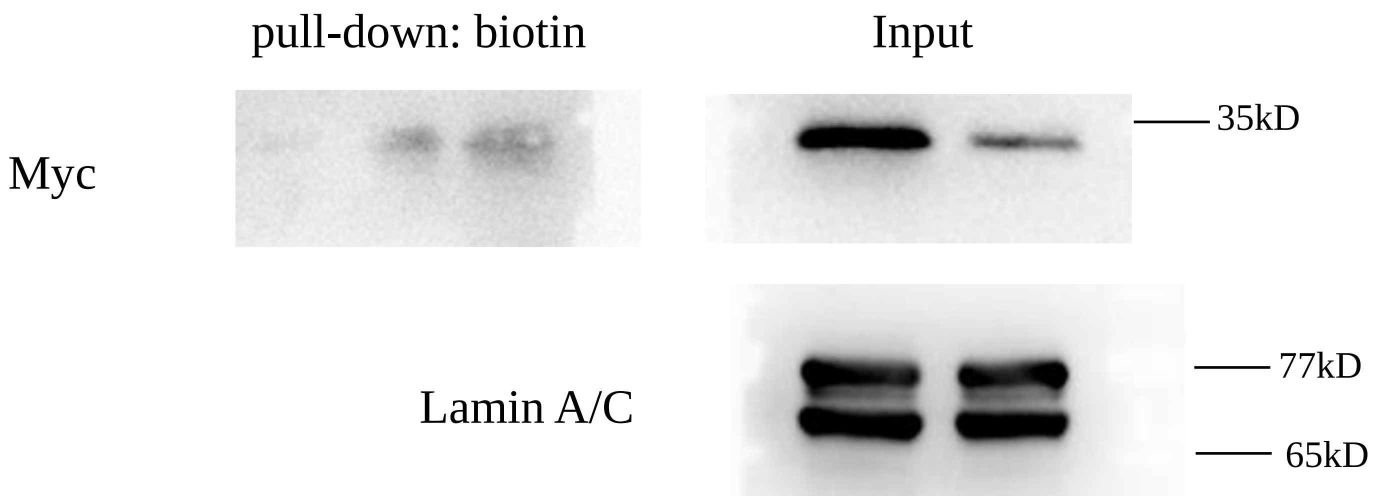

Figure 5C

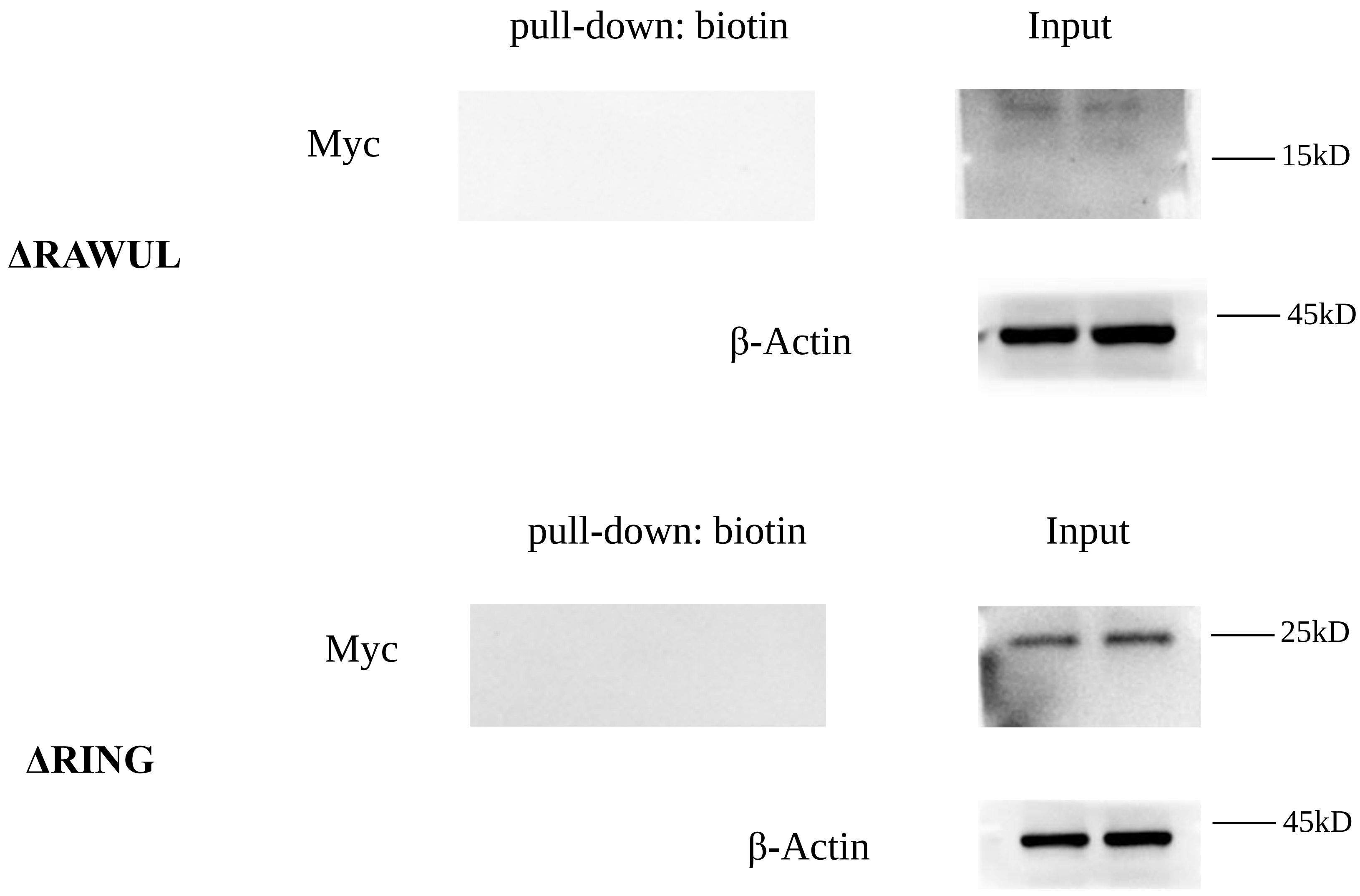
pull-down: biotin
Input
Myc
15kD
ΔRAWUL
β-Actin
45kD
pull-down: biotin
Input
Myc
25kD
ΔRING
β-Actin
45kD

Figure 6F

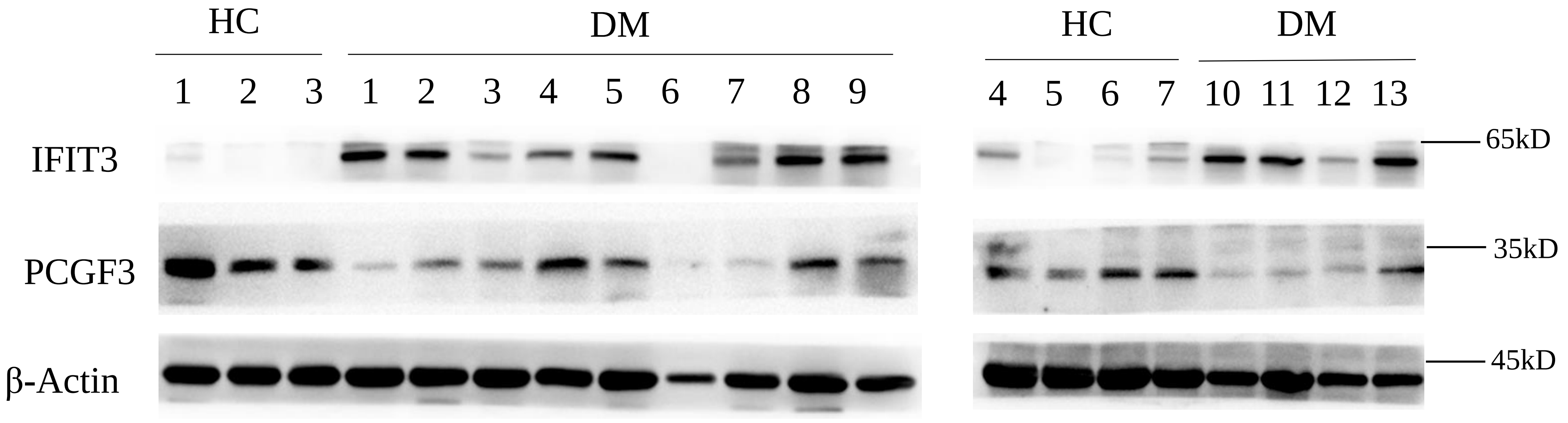

HC
DM
1 2 3 1 2 3 4 5 6 7 8 9
HC
DM
4 5 6 7 10 11 12 13
IFIT3
PCGF3
β-Actin
65kD
35kD
45kD

Figure S2C

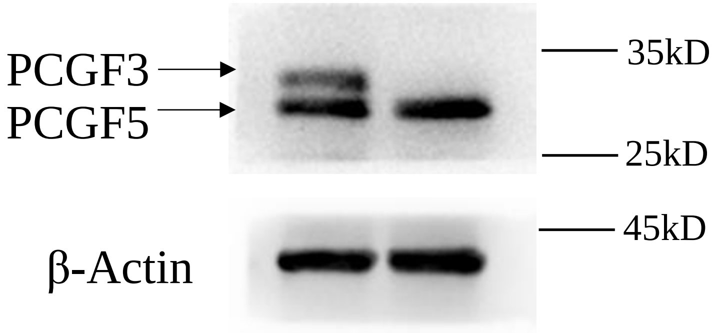

Supplement: Supplementary file 2 — Original Data [file 41420_2024_2194_MOESM2_ESM.pdf]
